# Development of a Blocking ELISA for Detection of Serum Neutralizing Antibodies against Newly Emerged Duck Tembusu Virus

**DOI:** 10.1371/journal.pone.0053026

**Published:** 2012-12-31

**Authors:** Xuesong Li, Guoxin Li, Qiaoyang Teng, Lei Yu, Xiaogang Wu, Zejun Li

**Affiliations:** Shanghai Veterinary Research Institute, Chinese Academy of Agricultural Sciences, Shanghai, People’s Republic of China; The Ohio State University, United States of America

## Abstract

**Background:**

Since April 2010, domesticated ducks in China have been suffering from an emerging infectious disease characterized by retarded growth, high fever, loss of appetite, decline in egg production, and death. The causative agent was identified as a duck Tembusu virus (DTMUV), a member of the Ntaya virus (NTAV) group within the genus *Flavivirus*, family *Flaviviridae*. DTMUV is highly contagious and spreads rapidly in many species of ducks. More than 10 million shelducks have been infected and approximately 1 million died in 2010. The disease remains a constant threat to the duck industry; however, it is not known whether DTMUV can infect humans or other mammalians, despite the fact that the virus has spread widely in southeast China, one of the most densely populated areas in the world. The lack of reliable methods to detect the serum antibodies against DTMUV has limited our ability to conduct epidemiological investigations in various natural hosts and to evaluate the efficiency of vaccines to DTMUV.

**Methodology/Principal Findings:**

A neutralizing monoclonal antibody (mAb) 1F5 binding specifically to the E protein was developed. Based on the mAb, a blocking enzyme-linked immunosorbent assay (ELISA) was developed for the detection of neutralizing antibodies against DTMUV. The average value of percent inhibition (PI) of 350 duck serum samples obtained from DTMUV-free farms was 1.0% ±5.8% (mean ± SD). The selected cut-off PI values for negative and positive sera were 12.6% (mean +2SD) and 18.4% (mean +3SD), respectively. When compared with a serum neutralizing antibody test (SNT) using chicken embryonated eggs, the rate of coincidence was 70.6% between the blocking ELISA and SNT, based on the titration of 20 duck DTMUV-positive serum samples.

**Conclusions/Significance:**

The blocking ELISA based on a neutralizing mAb allowed rapid, sensitive, and specific detection of neutralization-related antibodies against DTMUV.

## Introduction

Since April 2010, an outbreak of an infectious disease has spread widely throughout most of the domestic duck population in China, resulting in retarded growth, high fever, loss of appetite, decline in egg production, and death of the birds [1]. The causative agent of the disease was identified as a newly emerged duck Tembusu virus (DTMUV). The outbreak was first detected in Shanghai, but spread rapidly to all of the southeast provinces of China, including Zhejiang, Jiangsu, Fujian, and Anhui, and the transmission of the virus persisted until the winter season. Shelducks in the provinces of Shandong, Henan, Hunan, Hubei, and Jiangxi were particularly susceptible to this virus, with 100% infection, and morbidity rates and mortality rates ranging from 5% to 30%. To date, more than 10 million shelducks have been infected, and approximately 1 million have died. DTMUV has also been recently isolated from other poultry such as geese and sparrows [2], [3].

Continuous surveillance is the main approach to collect and analyze DTMUV epidemic information. Several diagnostic methods for DTMUV detection have been reported, including virus isolation assays [1], reverse-transcription polymerase chain reaction (RT-PCR) [4], real-time PCR [5], and reverse-transcription loop-mediated isothermal amplification assay [6], [7]. Because DTMUV is no longer detectable after the infected poultry recover, serological surveillance is more important than detection of virus to monitor DTMUV prevalence. At present, no rapid, sensitive, and specific method exists to detect antibodies against DTMUV.

To develop the specific methods to detect antibodies against DTMUV, we established a blocking ELISA method for detecting the neutralizing antibodies against DTMUV, as detailed in this study.

## Materials and Methods

### Viruses

Duck Tembusu virus FX2010 isolated from sick shelducks in China [1] was propagated on DF-1 cells. The cell debris was removed by centrifugation at 7,500 rpm for 30 min at 4°C, and the virus was deactivated with 2% formaldehyde as described previously [8]. The virus particles were pelleted by ultracentrifugation and used as immunization antigens for antibody production or coating antigens for blocking ELISA.

### Development of mAbs Against DTMUV

The mAbs against DTMUV were produced as described previously [9]. Briefly, 6-week-old female SPF BALB/c mice were immunized subcutaneously with 50 µg of purified DTMUV mixed with complete Freund’s adjuvant, and boosted 3 times with the same dose every 2 weeks. Finally, the mice were injected subcutaneously with the same dose of DTMUV without any adjuvant 4 weeks after the final immunization. Three days following administration of the final booster, the mice were euthanized using sodium pentobarbital (Sigma**)**, and their spleen cells were isolated and fused with SP2/0 using 50% polyethylene glycol (Sigma, USA). The fused cells were seeded in 96-well plates and cultured in hypoxanthine–aminopterin–thymidine (HAT) selective medium. The monoclonal antibodies secreted by hybridomas were screened by indirect ELISA using 96-well polystyrene flat-bottomed microtiter plates coated with the inactive DTMUV purified by ultra-centrifugation through 40% w/v sucrose. The hybridomas were sub-cloned 3 times by the limiting dilution method in 96-well plates (Costar Corning Inc., Corning, NY, USA) [10]. The mAbs were classified using a SBA Clonotyping™ System/HRP kit (SouthernBiotech, USA). The mouse studies were approved by the Animal Care and Use Committee of Shanghai Veterinary Research Institute, Chinese Academy of Agricultural Sciences.

### Immunofluorescence Assay (IFA)

The activity of the mAbs was tested by Immunofluorescence assays as described previously (IFA) [11]. Briefly, the DF-1 cells on 6-well plates were incubated with 10^4.5^ TCID_50_ DTMUV (FX-2010) in 1 mL of phosphate-buffered saline (PBS) for 1 h at 37°C. Meanwhile, the normal DF-1 cells were used as negative controls. After the cells were washed 4 times with PBS to remove surplus virus, fresh medium was added and the cells were incubated at 37°C. At 36 h post-infection, the cells were fixed with 4% paraformaldehyde for 20 min at room temperature and washed again. After being blocked with 10% BSA for 20 min at room temperature, the cells were incubated with monoclonal antibody 1F5 for 30 min. Cells were then incubated with fluorescein isothiocyanate-labeled goat anti-mouse antibody immunoglobulin G (IgG, 1∶200 dilution; Sigma, USA), for 30 min, washed, and mounted with 10 mM PPD (p-phenylenediamine) in glycerol:PBS (9∶1), pH 8.5. Samples were observed under a fluorescent microscope.

A recombinant eukaryotic expressing plasmid (pCAGGs-TMUV-E) was generated by inserting the open reading frame (ORF) of the E gene of FX2010 into multiple cloning sites on the pCAGGs plasmid. 293T cells were transfected with pCAGGs-TMUV-E. Approximately 48 h post-transfection, the 293T cells were fixed with 4% paraformaldehyde for 20 min at room temperature and washed. An indirect immunofluorescence assay was conducted using the monoclonal antibody 1F5 as described previously, and cells were examined under a fluorescence microscope.

### Prokaryotic Expression of DTMUV E Protein Domain III

The recombinant prokaryotic expression plasmid (pCold-EDIII) expressing the fusion protein containing DTMUV E protein domain III and trigger factor (TF) tag protein in *E. coli* BL21 cells, was constructed in our lab. After the *E. coli* BL21 transformants containing pCold-EDIII were induced with isopropyl- thiogalactopyranoside (IPTG) for 4 h, the cultural solution was centrifuged at 7,500 g for 10 min at 4°C. The resulting bacterial pellet was then suspended and lysed by sonication. The fusion protein was purified from soluble proteins using a commercial protein purification product (Ni-NTA His·Bind Resin, Novagen, Madison, USA) and stored at −20°C.

### Western Blot Analysis

For western blot analysis, 50 µg of the purified fusion protein, including both the E protein domain III and TF tag protein, and purified TF tag protein expressed by pCold plasmids (Takara, Dalian, P. R. China), were used for sodium dodecyl sulfate polyacrylamide gel electrophoresis (SDS-PAGE). The separated proteins were then electroblotted onto a polyvinylidene fluoride membrane and blocked with 5% skimmed milk in PBST. Following incubation with 1F5, the membrane was rinsed with PBST and incubated with horseradish peroxidase (HRP)-conjugated goat anti-mouse immunoglobulin G (IgG) (Sigma, USA) for 1 h at 37°C. The membrane was subsequently analyzed with a chemiluminescent substrate (ECL, Thermo scientific, Pierce, USA).

### Duck Sera

Anti-DTMUV duck sera were collected from experimentally infected shelducks 2 weeks after they were inoculated intranasally with 10^5.5^ TCID_50_ FX2010. The duck studies were approved by the Animal Care and Use Committee of Shanghai Veterinary Research Institute, Chinese Academy of Agricultural Sciences. Twenty duck sera with 4 different blocking ELISA titers of anti-DTMUV antibody were selected from farm-raised ducks naturally infected by DTMUV. Sixty field serum samples collected from six duck farms were used to test the suitability of the blocking ELISA for field use. Negative sera were collected from non-infected shelducks. Anti-serum against H5N1 avian influenza virus (AIV), H9N2 AIV, Newcastle disease virus (NDV), type I duck hepatitis virus (DHV-1), duck plague virus (DPV), reovirus (RV), and Japanese encephalitis virus (JEV) were acquired by the Shanghai Veterinary Research Institute, and used to test the specificity of blocking ELISA.

### Serum Neutralizing Antibody Test (SNT)

The neutralization test (SNT) was performed on 8-day-old SPF chicken embryonated eggs as previously described [12]. Briefly, the serum samples deactivated at 56°C and the monoclonal antibodies were initially diluted 5-fold with PBS, then further diluted through a series of 2-fold dilutions. The diluted sera were mixed with 100 ELD_50_/0.1 mL of FX2010 at a volume ratio of 1∶1 and incubated at 37°C for 1 h. The virus-serum mixtures (200 µL) were inoculated into the allantoic cavity of 8-day-old SPF chicken embryonated eggs. PBS and negative serum were used as negative controls. Five days after incubation, the neutralization titers of sera were calculated by the Reed-Muench method [13].

### Development of Indirect ELISA

Indirect ELISA assay was used to assess the titers of DTMUV-specific antibody. Briefly, ELISA plates (Corning, USA) were coated with the purified fusion protein containing E protein domain III and TF tag protein (0.1 µg per well) and incubated overnight at 4°C. After blocking of the plates, test serum was added at a starting dilution of 1∶10, followed by the addition of 2-fold dilutions. HRP-conjugated goat anti-duck IgG (KPL, USA) was used to detect bound antibodies for 1 h at 37°C. The wells were rinsed with PBST and incubated with TMB. Substrate development was stopped by the addition of 0.1 N sulfuric acid, and the optical density (OD) was measured at 450 nm. The OD of each serum was expressed as the ratio of OD_450_ of a sample to that of a negative control (P/N) calculated based on the negative control serum in each microplate, in order to minimize variation between plates. The P/N was calculated according to the formula: P/N = OD test serum/OD negative control serum. The cut-off point was calculated based on the arithmetic mean of the P/N of the 350 sera samples found negative for neutralizing antibodies (mP/N), plus 3 standard deviations (s). Thus, the Cut-off point = mP/N +3s.

### Development of Blocking ELISA

Optimal dilutions of coating antigen and mAb 1F5 were determined by checkerboard titration. After the condition was optimized, ELISA plates were coated with approximately 3 µg/well purified FX2010 in 0.1 M carbonate–bicarbonate buffer (pH 9.6) and incubated overnight at 4°C. Antigen-coated plates were washed with PBS (pH 7.4) containing 0.05% Tween-20 (PBST), and the nonspecific binding sites were blocked with 100 µL of blocking buffer (PBS containing 5% skim milk) for 1 h at 37°C. Serum samples were initially diluted 10-fold with PBS, and then further diluted through a series of 2-fold dilutions. Aliquots (100 µL) of diluted serum were added to each well and incubated for 1 h at 37°C. The wells were then washed 3 times with PBST and incubated with mAb 1F5 (20×) for 1 h at 37°C. After the wells were rinsed with PBST 3 times, goat anti-mouse IgG (Sigma, USA) conjugated to HRP was added, and samples were incubated at room temperature for 1 h. After the wells were rinsed with PBST 3 times, 100 µL of 3,3′,5,5′-tetramethyl benzidine was added and cells were incubated at room temperature for 5 min. The reaction was then stopped by adding 0.1 N sulfuric acid. The optical density (OD) was measured at 450 nm, and the percent inhibition (PI) value was determined using the formula: PI (%) = (1 − (OD_450 nm_ of test serum/OD_450 nm_ of negative control serum)) × 100%. We used 350 duck sera samples from DTMUV-free farms for determining the cut-off value between the positive and negative sera samples. The cut-off value was designed as the mean PI of negative sera +2 or 3 standard deviations (SD), which would ensure that either 95% or 99% of PI values for the negative sera sample fell within this range. Each plate contained diploid positive and negative controls.

### Comparison of Indirect ELISA, Blocking ELISA and SNT

To test the coincidence of different methods, the antibody titers of 20 duck serum samples from recovered ducks infected by DTMUV were determined respectively by indirect ELISA, blocking ELISA and SNT. Briefly, the serum samples were initially diluted 5-fold with PBS, then further diluted through a series of 2-fold dilutions. The diluted samples were used in indirect ELISA, blocking ELISA and SNT. The SNT titers were calculated from the living embryos in different dilutions by the Reed-Muench method as described in Methods. The blocking ELISA titers and indirect ELISA titers corresponded to the highest dilution factor that still yields a positive reading in blocking ELISA titers and in indirect ELISA respectively. Coincidence rates between different methods were calculated using Microsoft Excel's CORREL function.

### Isolation of DTMUV from Duck Serum

Aliquots (100 µL) of undiluted serum were added onto the monolayer DF-1 cells cultured on 6-well plates. After incubation for 2 h at 37°C, the cells were washed 2 times with PBS and DMEM medium with 2% fetal serum was added. The cells were cultured continually at 37°C with 5% CO_2_ for 72 h, and then the supernatant was collected and the cells were fixed with 4% paraformaldehyde for 20 min at room temperature. To verify DTMUV infection, the fixed cells were analyzed by the IFA using monoclonal antibody 1F5 as described above.

### Field Application of Blocking ELISA

The serum samples collected from 6 duck farms were used for the DTMUV-specific antibodies detection. The blocking ELISA and SNT were performed as described previously. Virus isolation was conducted to determine whether the ducks were suffering from DTMUV infection when DTMUV-specific antibodies from different ducks varied by large deviations on a single farm.

## Results

### Characterization of mAbs

A total of 6 antibodies against DTMUV secreted by the monoclonal hybridomas were selected by indirect ELISA using 96-well polystyrene flat-bottomed microtiter plates coated with the purified DTMUV.

**Figure 1:**
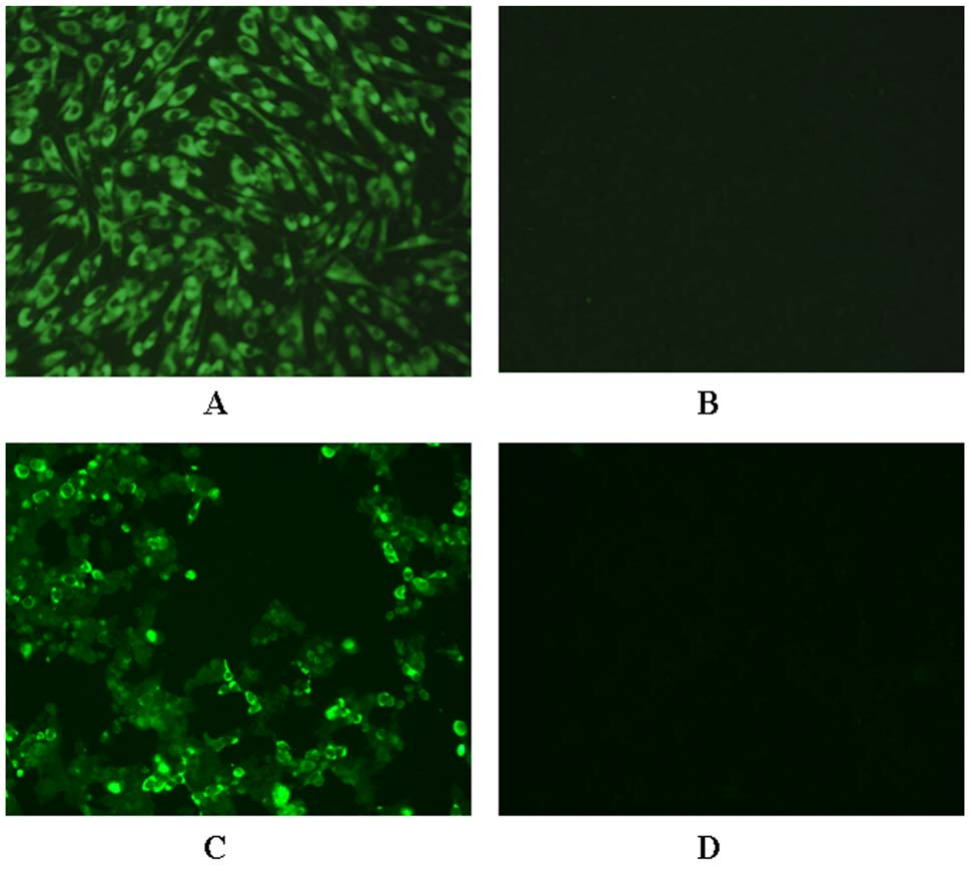
Characterization of monoclonal antibody 1F5 by immunofluorescence assay. Monoclonal antibody 1F5 was used to perform indirect immunofluorescence assay on DF-1 cells infected with DTMUV FX2010 and 293T cells transfected with pCAGGS-E plasmids. A) DF-1 cells infected with DTMUV FX2010, B) control DF-1 cells, C) 293T cells transfected with recombinant plasmid pCAGGS-E, and D) control 293T cells fixed with 4% paraformaldehyde, and then incubated with mAb 1F5 and FITC-conjugated goat anti-mouse IgG, in turn. Cells were mounted with 10 mM p-phenylenediamine (PPD) in glycerol-PBS and observed under a fluorescent microscope.

To determine the specificities of the monoclonal antibodies (mAbs), DF-1 cells infected with FX2010 and 293T cells transfected with the recombinant eukaryotic expressing plasmid (pCAGGs-TMUV-E) expressing E protein of DTMUV were used for IFA. Only the mAb 1F5 yielded immunofluorescence in the cytoplasm of DF-1 cells infected with FX2010 virus (Fig. 1 A and B). Moreover, 1F5 yielded immunofluorescence in the cytoplasm of 293T cells transfected with pCAGGs-TMUV-E (Fig. 1 C and D).

**Figure 2:**
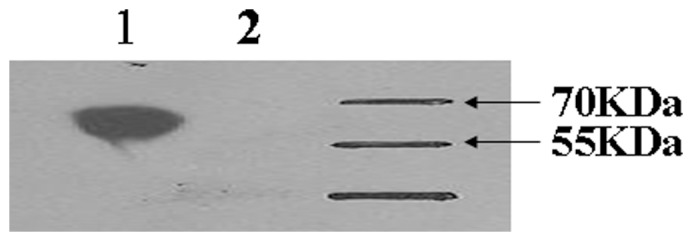
Specificity of the b-ELISA to anti-DTMUV serum. To test the abilities of mAb to bind specifically to domain III of E protein, western blot was conducted with purified fusion protein including both the domain III (12 kDa) of E protein and TF tag protein (52 kDa) (line 1), and purified TF tag protein (52 kDa) (line 2) expressed by pCold plasmids. The mAb 1F5 was able to bind specifically to the 64-kDa fusion protein, but could not bind to purified TF tag protein (Fig 2).

To test the abilities of mAb to bind specifically to domain III of E protein, western blot was conducted with purified fusion protein including both the E protein domain III (12 kDa) and TF tag protein (52 kDa), and purified TF tag protein (52 kDa) expressed by pCold plasmids. The mAb 1F5 was able to bind specifically to the 64-kDa fusion protein, but not to the purified 52-kDa TF tag protein (Fig. 2).

To test whether 1F5 could neutralize DTMUV, the neutralization test (SNT) was performed on 8-day-old SPF chicken embryonated eggs. The neutralizing activity of 1F5 against DTMUV was confirmed and the neutralizing titer was 40. The isotype of 1F5 was identified as IgG2a class by Clonotyping™ System/HRP kit.

### Development of the Indirect ELISA Assay

Indirect ELISA assay was established using the purified fusion protein containing the domain III of E protein and TF tag protein. To determine the cut-off values of P/N in indirect ELISA, a panel of 350 duck serum samples from DTMUV-free farms was used. The average P/N value of those negative sera was 1.3±0.3. Accordingly, the Cut-off point of the indirect ELISA was 2.2.

### Development of the Blocking ELISA Assay

A blocking ELISA assay was established based on the ability of anti-DTMUV serum to block the neutralizing binding site targeted by 1F5. To determine the cut-off values of PI in blocking ELISA, a panel of 350 duck serum samples from DTMUV-free farms was used. The average PI value of those negative sera was 1.0% ±5.8%. Accordingly, the 95% and 99.7% confidence intervals for the PI values of negative sera ranged from −10.6% to 12.6% and from −16.4% to 18.4% respectively. The serum was considered positive to DTMUV when the PI value was ≥18.4%. When the PI value was ≤12.6%, the serum was negative. When the PI value was between 12.6% and 18.4% (12.6% <PI <18.4%), the serum was considered to be borderline. Repeated analyses were performed on the sera whose PI values were between 12.6% and 18.4%, and the sera were considered negative when the values were less than 18.4%.

### Specificity and Sensitivity of Blocking ELISA

**Figure 3:**
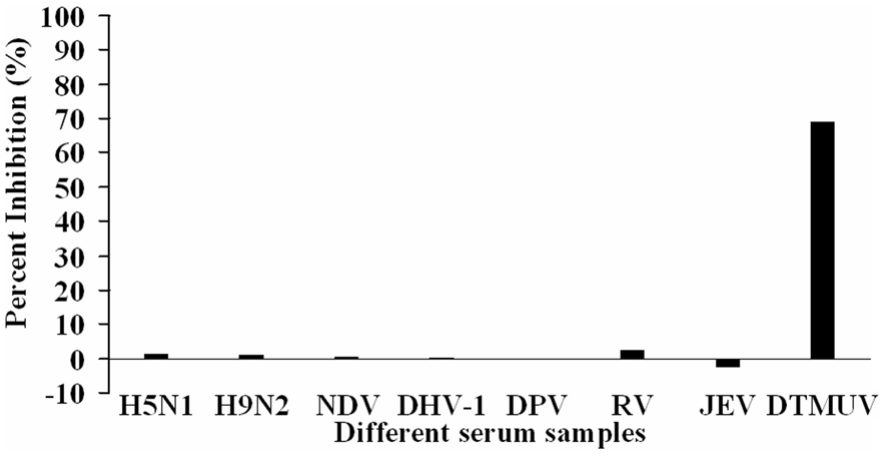
Specificity of the b-ELISA to anti-DTMUV serum. Seven antisera against different viruses were investigated. The PI value of anti-DTMUV serum reached a maximum of 69.13%, while the other antisera against H5N1 AIV, H9N2 AIV, NDV, DHV-1, DPV, RV, and JEV ranged from −2.7% to 2.3%.

To test the specificity of blocking ELISA, different antisera against the potential duck-infecting viruses were investigated. The PI value of anti-DTMUV serum reached a maximum value of 69.13%, while the PI values of antisera against H5N1 AIV, H9N2 AIV, NDV, DHV-1, DPV, RV, and JEV were 1.4%, 1.2%, 0.5%, 0.13%, −0.2%, 2.3%, and −2.7%, respectively. This indicates that the blocking ELISA was specific to detect the antibody against DTMUV and did not cross-react with other antisera (Fig. 3).

The sensitivity of the blocking ELISA was compared to SNT and evaluated using diluted anti-DTMUV serum. The maximal detectable dilution in blocking ELISA was 2-fold higher than in SNT, indicating that the blocking ELISA was more sensitive than SNT (Table 1).

**Table 1:** Comparison of percent inhibition obtained in blocking ELISA and SNT using a serially diluted duck anti-DTMUV serum.

Serum dilution	PI (%)	SNT
1∶2	76.71 (+)	+
1∶4	70.90 (+)	+
1∶8	64.36 (+)	+
1∶16	54.37 (+)	+
1∶32	40.93 (+)	+
1∶64	29.32 (+)	−
1∶128	15.65 (−)	−
1∶256	−0.88 (−)	−

Note: “+” positive and “−” negative.

### Correlative Analysis of Serum Antibody Titers Determined by Indirect ELISA, by Blocking ELISA and by SNT

To compare serum antibody titers determined by indirect ELISA, by blocking ELISA and by SNT, 20 duck sera samples with different blocking ELISA titers of anti-DTMUV antibody were selected from recovered farm-raised ducks infected by DTMUV. The sera were diluted and tested, respectively, as described in the Methods section. The neutralization titers determined by SNT were calculated from the living embryos in different dilutions by the Reed-Muench method [13]. The blocking ELISA titers and indirect ELISA titers corresponded to the highest dilution factor that still yielded a positive reading in blocking ELISA titers and in indirect ELISA, respectively. Results revealed a positive coincidence between the blocking ELISA and the SNT, with a coincidence rate of 70.6% (Table 2). The coincidence rate between indirect ELISA and blocking ELISA was 0.35, and the coincidence rate between indirect ELISA and SNT was 0.44.

**Table 2:** Results of B-ELISA, I-ELISA and SNT with reference sera.

Samples	Blocking ELISA titer	Indirect ELISA titer	SNT titer
1	10	80	7.07
2	10	80	5.95
3	10	10	7.07
4	10	80	5.95
5	10	10	8.41
6	20	20	20
7	20	40	14.15
8	20	80	11.89
9	20	80	7.07
10	20	40	11.89
11	40	80	23.78
12	40	80	24.62
13	40	160	10
14	40	160	25.61
15	40	40	20
16	80	160	46.3
17	80	40	14.59
18	80	80	31.09
19	80	80	20
20	80	80	20

Note: Coincidence rates between different methods were calculated using Microsoft Excel's CORREL function. The results showed that the blocking ELISA and the SNT have a positive coincidence with coincidence rate of 70.6%. The coincidence rate between indirect ELISA and blocking ELISA was 0.35, and the coincidence rate between indirect ELISA and SNT was 0.44.

### Field Application of Blocking ELISA

To test the suitability of the blocking ELISA for field use, 60 sera samples collected from 6 duck farms were used. Of these, 34 sera samples exhibited positive tests by the blocking ELISA, while 33 were SNT positive. The positive tests originated from only 4 of the 6 farms, with positive rates of 100% from 3 farms and 40% for the fourth farm (Table 3). In addition, because the DTMUV-specific antibodies from different ducks varied significantly from farm D (Table 3), a virus isolation was conducted on DF-1 cells. The DTMUV was isolated from 4 of 10 sera samples and confirmed that the DTMUV infections were occurring in the ducks from this farm.

**Table 3:** Results of testing field –origin duck sera in blocking ELISA and SNT.

Farms	samples	PI in blocking ELISA a	SNT titer b
A	A-1	67.4% (+)	≥5 (+)
	A-2	69.0% (+)	≥5 (+)
	A-3	66.3% (+)	≥5 (+)
	A-4	77.1% (+)	≥5 (+)
	A-5	74.6% (+)	≥5 (+)
	A-6	77.0% (+)	≥5 (+)
	A-7	70.3% (+)	≥5 (+)
	A-8	65.7% (+)	≥5 (+)
	A-9	66.0% (+)	≥5 (+)
	A10	72.8% (+)	≥5 (+)
B	B-1	68.3% (+)	≥5 (+)
	B-2	69.3% (+)	≥5 (+)
	B-3	78.2% (+)	≥5 (+)
	B-4	68.4% (+)	≥5 (+)
	B-5	74.5% (+)	≥5 (+)
	B-6	72.7% (+)	≥5 (+)
	B-7	62.9% (+)	≥5 (+)
	B-8	60.7% (+)	≥5 (+)
C	C-1	67.2% (+)	≥5 (+)
	C-2	64.0% (+)	≥5 (+)
	C-3	69.0% (+)	≥5 (+)
	C-4	70.0% (+)	≥5 (+)
	C-5	69.5% (+)	≥5 (+)
	C-6	73.6% (+)	≥5 (+)
	C-7	72.1% (+)	≥5 (+)
	C-8	66.4% (+)	≥5 (+)
	C-9	64.0% (+)	≥5 (+)
	C-10	64.4% (+)	≥5 (+)
	C-11	65.7% (+)	≥5 (+)
	C-12	54.8% (+)	≥5 (+)
D	D-1	52.6% (+)	≥5 (+)
	D-2	46.4% (+)	≥5 (+)
	D-3	4.6% (−)	<5 (−)
	D-4	−4.4% (−)	<5 (−)
	D-5	−5.7% (−)	<5 (−)
	D-6	28.5% (+)	<5 (−)
	D-7	−3.6% (−)	<5 (−)
	D-8	−4.2% (−)	<5 (−)
	D-9	50.2% (+)	≥5 (+)
	D-10	1.4% (−)	<5 (−)
E	E-1	−0.7% (−)	<5 (−)
	E-2	−1.9% (−)	<5 (−)
	E-3	3.5% (−)	<5 (−)
	E-4	−0.7% (−)	<5 (−)
	E-5	2.0% (−)	<5 (−)
	E-6	3.2% (−)	<5 (−)
	E-7	5.9% (−)	<5 (−)
	E-8	7.2% (−)	<5 (−)
	E-9	3.2% (−)	<5 (−)
	E-10	2.8% (−)	<5 (−)
F	F-1	3.6% (−)	<5 (−)
	F-2	8.4% (−)	<5 (−)
	F-3	−0.3% (−)	<5 (−)
	F-4	−5.9% (−)	<5 (−)
	F-5	3.0% (−)	<5 (−)
	F-6	1.2% (−)	<5 (−)
	F-7	−5.2% (−)	<5 (−)
	F-8	−0.3% (−)	<5 (−)
	F-9	1.3% (−)	<5 (−)
	F-10	8.0% (−)	<5 (−)

a Percent inhibition(PI) ≥18.4 was considered positive (indicated in brackets).

b SNT titers ≥5 were considered positive (indicated in brackets).

## Discussion

Duck viral disease caused by Tembusu viruses is an infectious disease that was first reported in 2010. The DTMUV infections can currently be diagnosed only within the first several days using tests that detect infectious particles and nucleic acids, therefore, active serological surveillance for DTMUV will be crucial for the detection and control of this emerging duck pathogen. Because serological identification of the infecting agent in *Flavivirus* infections is problematic due to the extensive cross-reactivity of *Flavivirus* antibodies [14], we therefore developed a blocking ELISA for the specific detection of serum antibodies against DTMUV based on a specific neutralizing monoclonal antibody against DTMUV.

Preliminary studies on *Flavivirus* have shown that the envelope protein E is the main structural protein. Protein E exhibits high immunogenicity and plays an important role in the activities of virus life cycles, such as binding to the receptor and invading host cells [15], [16]. The domain III of flavivirus E protein contains a panel of important epitopes that are recognized by virus-neutralizing mAbs. Peptides of the domain III have been used with promising results as antigens for flavivirus serologic diagnosis and as targets for immunization against these viruses [17]. In this study, to avoid the difficulties in prokaryotic expression of recombinant E protein, the recombinant domain III of FX2010 virus E protein, but not the recombinant E protein, was used to test the specificity of monoclonal antibodies and to establish the indirect ELISA.

Blocking ELISA, a specific method for antibody detection, has been used widely to diagnose human diseases [18], monitor animal infectious diseases [19], [20], [21], [22], [23], and detect viral antibodies in clinics or laboratories [24], [25], [26], [27], [28], [29]. The distinct advantages of blocking ELISA include high-volume sample testing, applicability across multiple species, and greater objectivity than some traditional techniques. Additionally, the assay requires only small volumes of sera. Since the isolation of DTMUV in ducks in 2010, several reports have described methods to detect DTMUV particles or its corresponding nucleic acids [1], [5], [6], [30]. In this report, we describe, for the first time, a reliable serum surveillance method. When compared with SNT, the blocking ELISA established in our laboratory exhibited high correlation with SNT, but with higher sensitivity and faster results. The blocking ELISA can be used to detect the antibodies against DTMUV in the serum samples from ducks and other avian species by using constant anti-mouse secondary antibodies, while the specific second antibodies against other species are necessary in indirect ELISA.

In summary, a sensitive and specific blocking ELISA has been established for the detection of DTMUV infection and the determination of the antibody titers against DTMUV in different avian species.
